# fMRI Guided rTMS Evidence for Reduced Left Prefrontal Involvement after Task Practice

**DOI:** 10.1371/journal.pone.0080256

**Published:** 2013-12-20

**Authors:** Johan Martijn Jansma, Tamar R. van Raalten, Ruud Boessen, Sebastiaan F. W. Neggers, Richard H. A. H. Jacobs, René S. Kahn, Nick F. Ramsey

**Affiliations:** 1 Department of Neurology and Neurosurgery, Rudolf Magnus Institute of Neuroscience, University Medical Centre Utrecht, Utrecht, The Netherlands; 2 Department of Child- and Adolescent Psychiatry, Rudolf Magnus Institute of Neuroscience, University Medical Centre Utrecht, Utrecht, The Netherlands; 3 Department of Psychiatry, Rudolf Magnus Institute of Neuroscience, University Medical Centre Utrecht, Utrecht, The Netherlands; University Medical Center Groningen UMCG, Netherlands

## Abstract

**Introduction:**

Cognitive tasks that do not change the required response for a stimulus over time (‘consistent mapping’) show dramatically improved performance after relative short periods of practice. This improvement is associated with reduced brain activity in a large network of brain regions, including left prefrontal and parietal cortex. The present study used fMRI-guided repetitive transcranial magnetic stimulation (rTMS), which has been shown to reduce processing efficacy, to examine if the reduced activity in these regions also reflects reduced involvement, or possibly increased efficiency.

**Methods:**

First, subjects performed runs of a Sternberg task in the scanner with novel or practiced target-sets. This data was used to identify individual sites for left prefrontal and parietal peak brain activity, as well as to examine the change in activity related to practice. Outside of the scanner, real and sham rTMS was applied at left prefrontal and parietal cortex to examine their involvement novel and practiced conditions.

**Results:**

Prefrontal as well as parietal rTMS significantly reduced target accuracy for novel targets. Prefrontal, but not parietal, rTMS interference was significantly lower for practiced than novel target-sets. rTMS did not affect non-target accuracy, or reaction time in any condition.

**Discussion:**

These results show that task practice in a consistent environment reduces involvement of the prefrontal cortex. Our findings suggest that prefrontal cortex is predominantly involved in target maintenance and comparison, as rTMS interference was only detectable for targets. Findings support process switching hypotheses that propose that practice creates the possibility to select a response without the need to compare with target items. Our results also support the notion that practice allows for redistribution of limited maintenance resources.

## Introduction

Performing a task for the first time typically is slow and prone to errors, while the amount of information that can be processed is limited. However, even relative short periods of practice can result in a striking improvement in performance if a task environment is consistent, in the sense that a certain stimulus always requires an identical response [1] [2,3]. Such tasks are also called “consistent mapping” (‘CM’) tasks. In contrast, if a task environment is inconsistent, and a stimulus may sometime require one response and at other ties another, practice may have almost no effect on performance [4]. These task are also referred to as ‘varied mapping’ (‘VM’) tasks.

Previous imaging studies have shown that practice CM tasks is also associated with a widespread reduction of brain activity [5-11]. FMRI studies are however limited in providing information about underlying causes of changes in brain activity. For instance, it is possible that reduced activity after practice reflects reduced involvement of these regions. It is however also possible that involvement of these regions hasn’t changed, but that the reduction is for instance a result of increased efficiency, or the loss of redundant activity.

Transcranial magnetic stimulation (TMS) can be used to briefly affect processing efficacy in a brain region, thereby revealing a causal relationship between cortical function and behavior [12]. For this reason, TMS is especially well suited to examine involvement of brain regions in task execution. Although the precise working mechanism of TMS is still somewhat unclear, a disruption of function is expected when the TMS induced current is remote from the operations that are normally performed by the neuronal tissue under investigation. This can for instance be achieved by inducing a train of repetitive short stimuli [13]. This technique, known as repetitive TMS (‘rTMS’), is understood to add noise to the intricate process of neuronal signaling, thereby reducing the processing efficacy in that brain region [12,14,15].

Here we present, to our knowledge, the first study that uses fMRI guided rTMS to examine the effect of practice on the involvement of specific brain regions. For this study, we focused on the left prefrontal and parietal cortex, as it has been shown that for the Sternberg task that we used [16], novel performance consistently induces robust activation in these regions, while practiced consistently reduces activity in these regions [7-9]. We used fMRI to identify a focus site for each subject within the left prefrontal and the left parietal cortex that showed maximal activity during novel performance. We then applied rTMS to those individual focus sites.

We hypothesized that the previously shown reduction of activity in left prefrontal and parietal cortex reflected reduced involvement of these regions. Based on this hypothesis, our expectation was that rTMS of the left prefrontal as well as left parietal cortex would interfere measurably with performance when the target-set was novel, as these regions are highly active in that condition. We expect that this interference would be significantly lower for practiced targets, as these regions are less active in this condition, and we hypothesize that this reduced activity reflects reduced involvement. 

## Materials and Methods

### Participants

Nineteen adult volunteers (10 male, 9 female; mean age = 22.8 years; sd = 3.03 years, range = 19-31 years) participated in the study after giving written informed consent. Subjects were recruited from the university campus through advertisement and rewarded for their participation. The Mini International Neuropsychiatric Interview (M.I.N.I.) [17] was used to exclude subjects with a history of neurological illness, psychiatric disorders, or substance abuse. All participants were tested for right-handedness using the Edinburgh Handedness Index [18]. The study was approved by the local medical ethics committee (‘Medisch-ethische toetsings commissie Universitair Medisch Centrum Utrecht’), in accordance with the Declaration of Helsinki (2008). Participants tolerated the rTMS protocol well and did not report any lasting adverse effects.

### Paradigm

We measured the difference in performance and brain activity associated with practice using a paradigm that had been successfully applied in previous studies [7-9]. The paradigm is based on a Sternberg task [16], but is specifically designed to register practice effects related to performing a consistent mapping task. For this purpose, the paradigm compares task performance of novel and practiced target sets. The two conditions are identical, except that subjects have had previous experience with the target-set in the practiced condition during a practice session. The training session is expected to induce improved performance [1], and reduced brain activity [7] for this specific target-set. 

We used a fixed set of ten consonants to create three target and non-target sets of five items for the practiced condition. One set was used during the fMRI session and the two other sets were used for the prefrontal and parietal rTMS sessions. We created all novel target-sets from the remaining ten consonants to prevent interference with the practiced target-sets. Novel target-sets were varied per block. 

### Task

**Figure 1:**
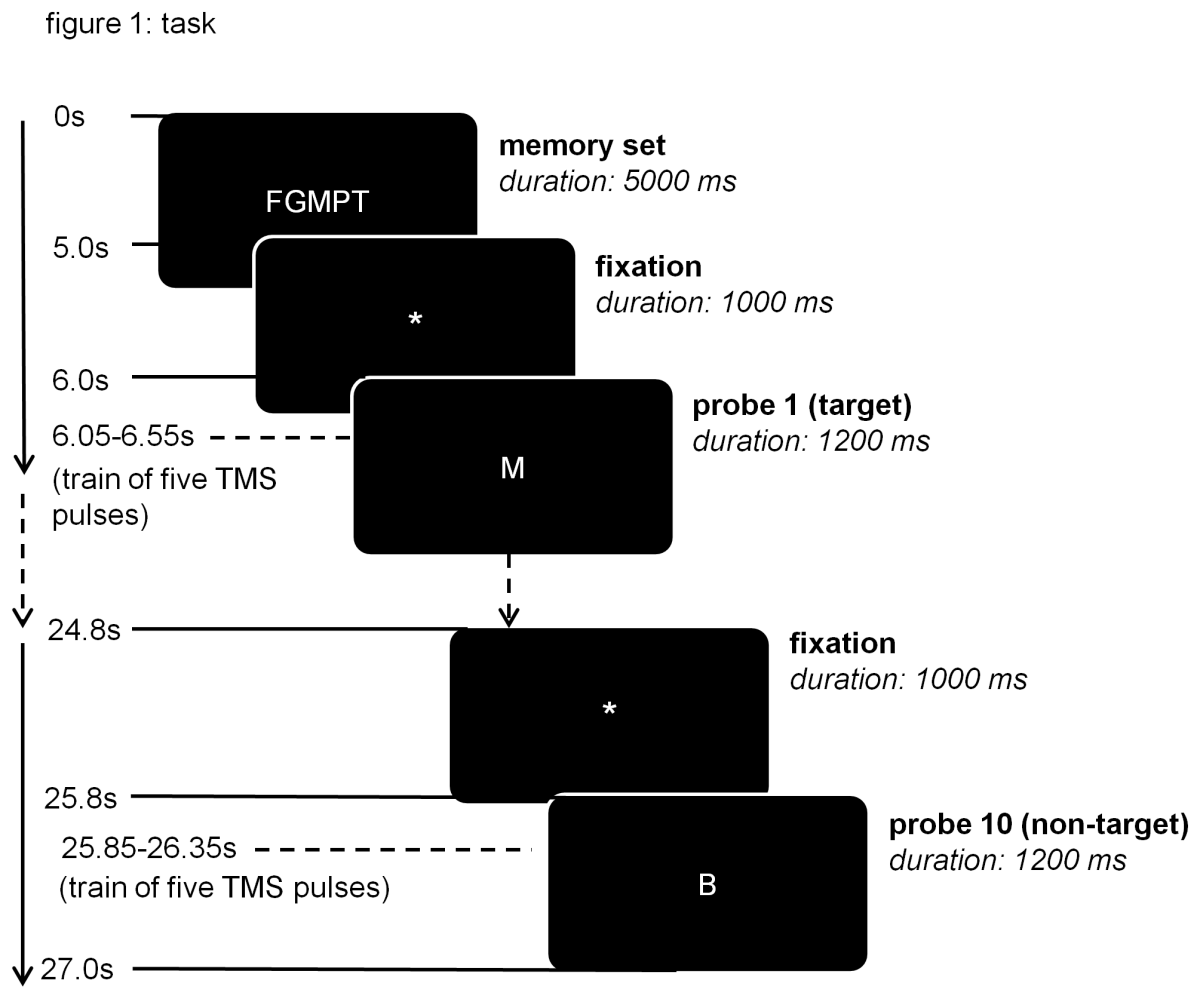
The temporal sequence is shown for the Sternberg task. Each run starts with the presentation a fixed memory set and is followed by ten probes. Subjects press a left button to targets and a right button to non-targets. For the novel condition, the target-set was varied for each run. For each practice run, the same target-set was used as in the practice session. For the baseline condition, the memory set consisted of two arrows (‘< ‘>’) and probe stimuli were single arrows (‘<’ or ‘>’). The task involved eight runs of each condition in a pseudorandom order. For the rTMS sessions, magnetic stimulation or sham stimulation was applied for 500 msec, starting 50 msec after the presentation of a probe.

We presented both the novel and practiced condition in runs of one target-set of five items, followed by ten probes (see Figure 1). Target-sets were presented for 5000 msec, probes were presented for 1200 msec and separated by an asterisk that was presented for 1000 msec. The occurrence of targets and non-targets was evenly distributed per run. We instructed subjects to memorize the target-set, and then use the right hand to press the left button of a pneumatic MRI compatible push-button box when probes matched the memory set (targets) or the right button if the probe did not match the memory set (non-targets). In the rTMS sessions, we instructed subjects to press the M on a QWERTY keyboard to targets and the X to non-targets. Both keys were clearly marked with an easily found ribbon to prevent searching during performance of the task. Subjects rested their left and right index fingers of the right hand on the keys during the entire session

Subject also performed a baseline condition, where subjects responded to the symbols ‘ <’ and ‘>’ by using the right hand to make a left or right button press respectively. The baseline condition required perceptual and motor processing, but no maintenance of a target-set. The baseline condition was used in the fMRI sessions to exclude perceptual and motor activity. All task versions used in the experiment were programmed in Presentation 9.9 running on a Windows operating system.

### Experimental procedure

**Figure 2:**
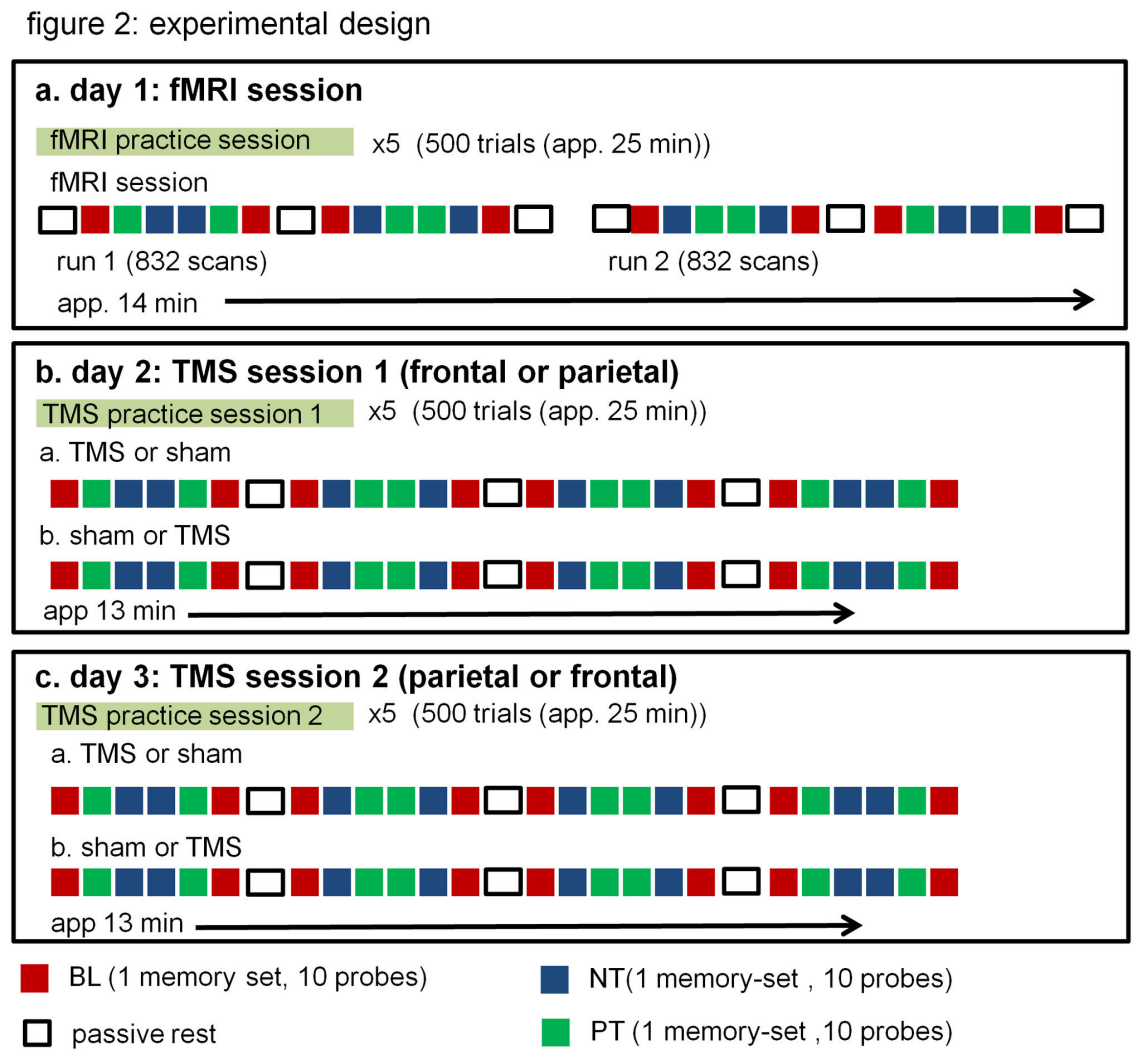
The experimental design. Subjects participated in one fMRI and two rTMS sessions, one for parietal and for prefrontal rTMS. Each session started with a practice session with a unique fixed target-set.

Subjects performed a scan session to determine individual target regions for TMS coil navigation, followed by two separate TMS sessions for the prefrontal and parietal stimulation sites (see Figure 2). In each of the two TMS sessions both a sham and real TMS stimulation was performed in random and balanced order. Each session was preceded by a training session that lasted approximately 25 minutes. In the training sessions, subjects performed the Sternberg task with a fixed target-set for 5 runs with 100 probes each. Each experiment session consisted of four sections separated by 32-second passive rest conditions. Each section started and ended with a baseline condition, and had two practiced and novel condition blocks in between, in balanced order for a total of 8 blocks of each condition in each session. 

### Functional MRI

#### FMRI data acquisition

FMRI was performed on a Philips 3 T Intera scanner. We reduced head movement by using a strap around the forehead and foam padding. A video projector located outside the scanner room projected the tasks on a 1m-wide through-projection screen, which subjects could view through a mirror attached to the head coil. Functional scans used a PRESTO SENSE pulse sequence image in two continuous runs of each 832 scans (parameters: TE = 32.4 msec, TR = 21.75 msec, voxel size = 4mm x 4mm x 4mm, 32 sagittal slice, scan duration of 500 msec) [19]. We acquired a T1-weighted anatomical image for spatial localization and to guide TMS coil navigation. 

#### FMRI analysis

**Figure 3:**
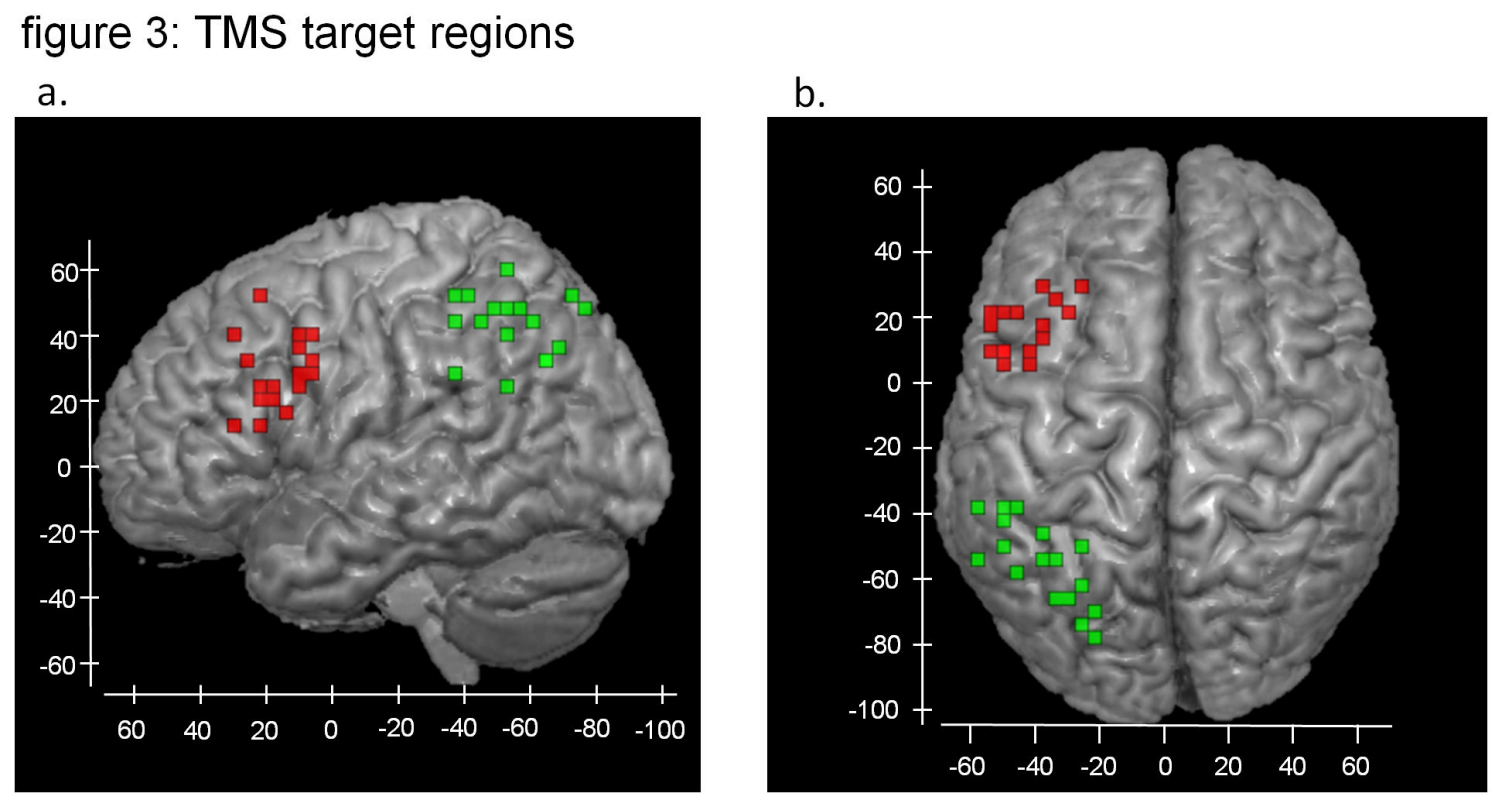
The individual stimulation locations in the left prefrontal (shown in red) and the left parietal cortex (shown in green) are displayed in MNI space for all participants in the study in a lateral (left) and a superior view (right). Individual stimulation locations were based on the voxel with the highest signal change in the novel-baseline contrast in the fMRI session. Numbers in blue represent MNI coordinates.

After reconstruction, functional and anatomical data were processed off-line using SPM5 software. Scans were corrected for motion, co-registered to the anatomy image, and spatially smoothed with a Gaussian kernel with FWHM of 8 mm. Individual statistical activation maps were generated in native space using a general linear model analysis. Separate regressors were used to model activity for the probe presentation period for each of the three conditions using a blocked paradigm approach [20,21]. We used the novel-baseline contrast t-maps in native space to identify rTMS focus site coordinates in the left prefrontal and the parietal cortex, based on the voxel with the maximum signal change. The individually derived rTMS target coordinates were also transformed into MNI space coordinates for visualization purposes (see Figure 3). 

In order to test if subjects showed reduced activity at each rTMS focus site, we calculated the fMRI signal for the novel and practiced condition at each of these positions. Additionally, we spatially normalized the activation maps to MNI space to create group activation patterns. These maps were used to examine regions showing a change in the signal after practice (activity threshold: |t| = 4.6, p < 0.0001 uncorrected), in order to test if there was any increase in activity in the practiced condition, compared to the novel condition. Presence of increased activity in the practiced condition could reduce the validity of our study, as it could mean that the focus site for rTMS that is chosen based on the novel condition, may not be valid for the practiced condition 

### TMS

#### Data acquisition

A frameless stereotactic neuronavigator (The Neural Navigator, http://www.neuralnavigator.com) from Brain Science Tools BV, the Netherlands, was used for coil positioning. (See 22 for details of the technique.) This device enables anatomical landmarks on the skin of the participants to be co-registered with the same landmarks on a skin rendering based on their MRI scans. We used the voxels with the highest signal change in the left prefrontal and parietal cortex in the novel-baseline fMRI contrast as rTMS target coordinates (see Figure 3). Each participant wore a tightly fitting swimming cap, where we marked the areas on the scalp directly overlying the rTMS target coordinates. Borders of the search areas were not strictly defined, but guided by the individual activation patterns. We refer to these regions as “frontal” and “parietal” for the remainder of the manuscript. 

We applied rTMS with a train of five rTMS pulses, separated by 100 msec (10 Hz). The pulses started 50 msec after the onset of each probe (see Figure 1) and covered the full response period for that probe, in order to reduce only the processing efficacy of that specific probe. A Neopulse TMS device (Neotonus Inc, Atlanta) with an iron core coil was used (see Epstein et al, 2002 for details). The iron core is embedded at the center of a rectangular figure of eight coil, parallel to the wiring at the center. Advantages of this coil are that the ferromagnetic cores cause the generated magnetic field to be stronger and to penetrate deeper into the brain (Epstein and Davey, 2002). The pulse intensity was 110% of the individual motor threshold, which was defined before the experiment as the minimum intensity that would induce a visible muscle twitch in the contra lateral hand on at least five out of ten occasions (see 23 for details). 

In order for us to control for nonspecific rTMS effects such as tactile and auditory sensations, participants also performed a session with a sham coil. The order of stimulation site (prefrontal and parietal) and session type (rTMS, sham) was balanced over subjects, to prevent a bias due to learning, fatigue, or habituation effects.

#### Data analysis

We obtained individual reaction time and accuracy data (proportion of correct responses) for each condition (novel, practiced, baseline), each site of stimulation (prefrontal, parietal), and each session (rTMS, sham). For the remainder of this manuscript, we refer to the difference in performance between the rTMS and sham session as the ‘rTMS effect’. 

### Hypotheses tests

For our hypotheses tests, we used the accuracy and reaction data for targets and non-targets for the prefrontal and parietal rTMS sessions. We determined all effects using a General Linear Model with univariate tests for repeated measures (SPSS17 ®). Effects of practice were tested using the main effects of condition (novel versus practiced). Effects of rTMS were tested using the main effect of stimulation (rTMS versus sham). Our main hypotheses, namely that the effect of rTMS would be smaller in the practiced condition than in the novel condition, was tested using the interaction effect of stimulation and condition. Furthermore we performed follow-up tests for the novel and practice condition separate.

## Results

### FMRI

**Figure 4:**
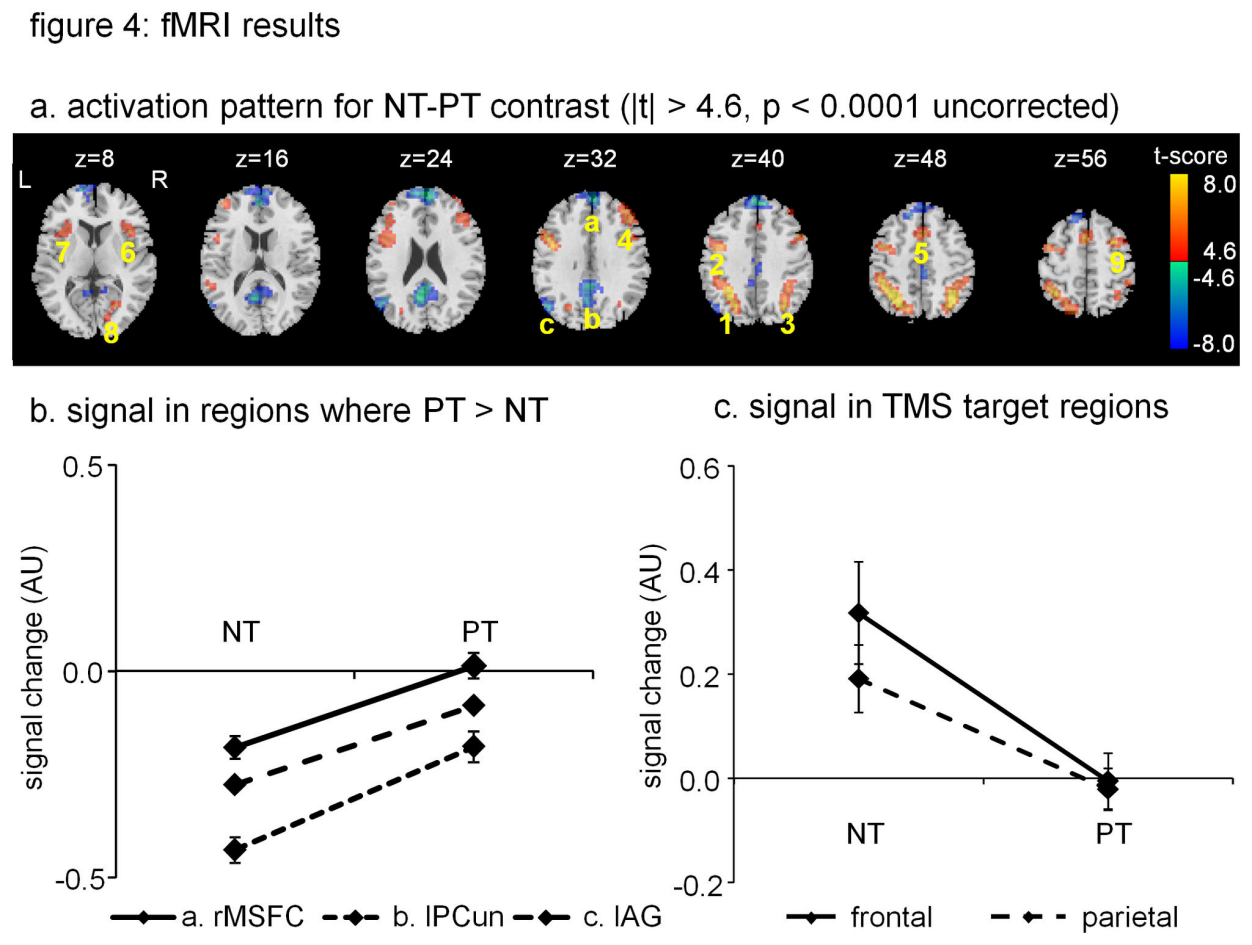
Summary of the fMRI results. a. regions showing significant difference between novel and practice condition (red: novel < practiced, blue: practiced > novel). Three regions showed an increase in activity for the practiced condition compare to the novel condition (a. right medial superior frontal cortex (rMSFC), b. left precuneus (lPCun), c. left Angular Gyrus (lAG). b. Signal change (baseline: rest) for the three regions where we found higher activity for practiced targets than for novel targets. The graph shows that in all three regions activity was below resting state activity for the novel condition, and closer to resting state for the practiced condition. Thus, none of these regions showed new or increased activity in the practiced condition, compared to the novel condition. c. fMRI signal measured at the individual prefrontal and parietal target regions for rTMS, based on the novel-baseline contrast. Both at the prefrontal and parietal regions, subjects showed significantly lower activity for practiced than for novel target-sets.

Individual stimulation locations in the prefrontal and parietal cortex in MNI space for each participant are displayed in Figure 3. Regions showing significantly different activity between the novel and practiced condition are summarized in table 1 and visualized in Figure 4a. As expected, practice significantly reduced activity in a network of regions including left inferior parietal, left precentral gyrus. In addition, there were three regions that showed increased activity: the medial part of the right superior frontal cortex, the left precuneus, and the left angular gyrus. Figure 4 illustrates that also in these three regions activity was closer to rest for the practiced condition than for the novel condition. 

**Table 1:** Overview of regions showing signal change in novel-practiced contrast.

ROI		description		abb.	BA	Size	MNI coordinates		
**novel > practiced**		**(AAL atlas)**				(cm^3^)	X	Y	Z
**1**		left inferior parietal gyrus		lIPG	40	16.6	-32	-52	44
**2**		left precentral gyrus		lPCG	6	12.2	-44	0	36
**3**		right angular gyrus		rAG	7	10.1	36	-56	48
**4**		right inferior frontal gyrus, triangular part		rIFGtri	45	5.2	44	32	28
**5**		left supplementary motor area		lSMA	6	5.0	-4	8	52
**6**		right insula		rINS	47	4.0	40	20	0
**7**		left insula		lINS	47	3.3	-36	20	0
**8**		right calcarine fissure		rCALC	18	3.1	16	-84	0
**9**		right middle frontal gyrus		rMFG	8	2.8	28	4	56
**practiced > novel**									
**a**		right superior frontal gyrus, medial part		rMSFG	9	19.2	8	48	40
**b**		left precuneus		lPCun	23	15.9	-4	-56	24
**c**		left Angular gyrus		lAG	39	4.3	-52	-68	28

MNI coordinates in table 1 refer to the voxel with maximum level which was also used to determine the name, using the AAL atlas [39]. Names should only be considered as descriptive with no anatomical meaning, as activity is based on a group average.

Figure 4c shows the average signal for the novel and practiced condition at the selected individual focus sites for the rTMS stimulation. Focus sites for rTMS showed a significant lower activity in the practiced than in the novel condition for both prefrontal (t = 5.53, p < 0.001) and parietal cortex (t = 5.42; p < 0.001). 

### rTMS and accuracy

#### Prefrontal rTMS session

**Figure 5:**
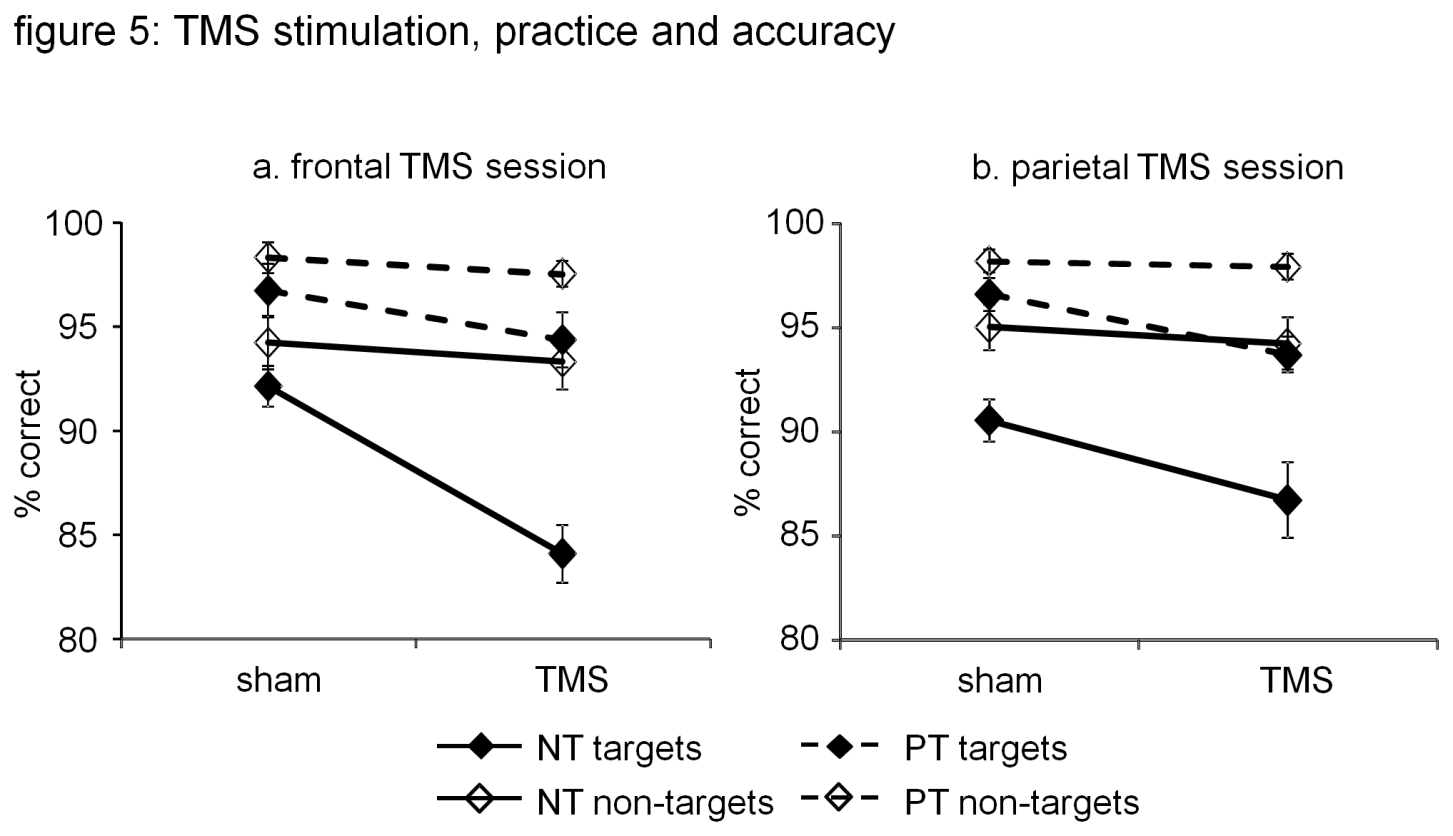
Effect of rTMS on accuracy. Graphs illustrate that practice increased accuracy for both targets and non-targets. Prefrontal rTMS reduced with target accuracy, and this effect was larger in the novel than in the practiced condition. Parietal rTMS also reduced target accuracy, but there was no difference between the novel and practiced condition. a. accuracy during prefrontal rTMS; b. accuracy during parietal rTMS.

Results are graphically displayed in Figure 5. For target accuracy, we found a significant main effect of condition (novel, practiced; F(1.18) = 62.05; p < 0.001) as well as a significant main effect of stimulation (rTMS, sham; F(1.18) = 16.13; p = 0.001). rTMS interference was significantly higher in the novel condition than in the practiced condition (F(1.18) = 9.04; p = 0.008). 

For non-targets, we also found a significant main effect of condition (novel, practiced; F(1.18) = 21.85; p < 0.001), but no main effect of stimulation (rTMS, sham; F(1,18) = 0.67; p = 0.43). Also, there was no difference in the prefrontal rTMS effect on non-target accuracy between the novel and practiced condition (F(1.18) = 0.007; p = 0.93)

#### Parietal rTMS session

For target accuracy, we also found a main effect of condition (novel, practice; F(1.18) = 33.26; p < 0.001), as well as a significant main effect of stimulation (rTMS, sham; F(1.18) = 9.40; p = 0.01). The parietal rTMS effect did not differ between the novel and practiced condition for target accuracy (F(1.18) = 0.21; p = 0.65).

For non-target accuracy, we also found a significant main effect of condition (novel, practiced; F(1.18) = 12.83; p < 0.001). We did not find a main effect of stimulation (rTMS, sham; F(1.18) = 0.57; p = 0.46), and no difference in parietal rTMS interference between the novel and practiced condition (F(1.18) = 0.21; p = 0.65)

### rTMS and reaction time

#### Prefrontal rTMS session

**Figure 6:**
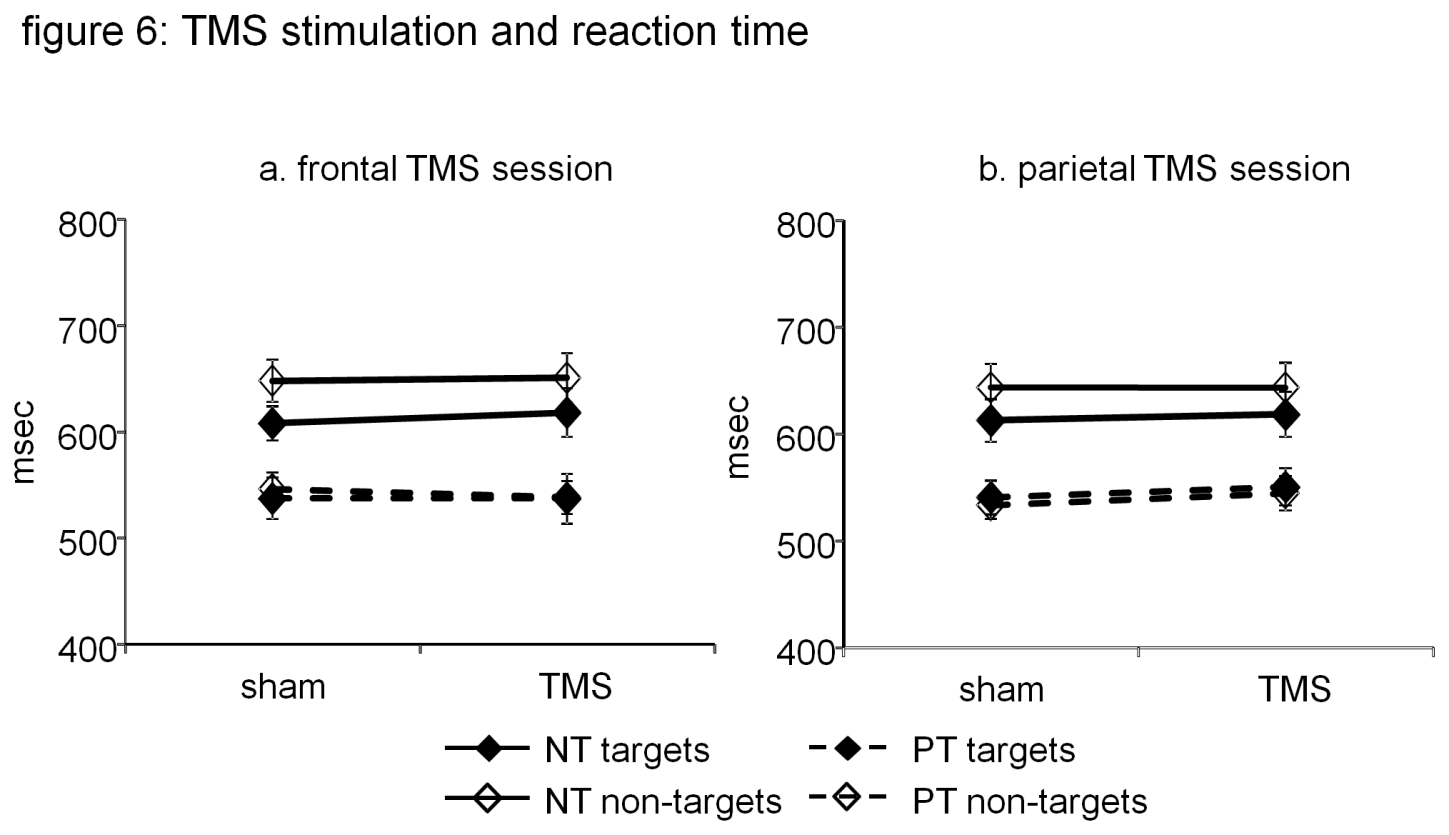
Effect of rTMS on reaction time. Graphs illustrate that reaction time was lower in the practiced than in the novel condition, but rTMS had no effect on reaction times. a. reaction time during prefrontal rTMS; b. reaction time during parietal rTMS.

Results are graphically displayed in Figure 6. For target reaction times, we found a significant main effect of condition (novel, practiced; F(1.18) = 29.32; p < 0.001). There was no main effect of stimulation (rTMS, sham; F(1.18) = 0.59; p = 0.45). Also, prefrontal rTMS effects were not different in the novel and practiced condition for target reaction times (F(1.18) = 1.12; p = 0.30). 

For non-target reaction times, we also found a significant main effect of condition (novel, practiced; F(1.18) = 42.72; p < 0.001), but no main effect of stimulation (rTMS, sham; F(1.18) = 0.015; p = 0.91). Also, there was no difference in prefrontal rTMS effects in the novel and practice condition for non-target reaction times (F(1.18) = 1.28; p =0.27)

#### Parietal rTMS session

For target reaction times, we found a significant main effect of condition (novel, practiced; F(1.18) = 61.2; p < 0.001), but no main effect of stimulation (rTMS, sham; F(1.18) = 0.12; p = 0.73). There was also no difference in parietal rTMS interference on target reaction times for the novel and practiced condition (F(1.18) = 0.04; p = 0.85).

For non-target reaction times, we also found a significant main effect of condition (novel, practiced; F(1.18) = 81.21; p < 0.001), no main effect of stimulation (rTMS, sham; F(1.18) = 0.58; p = 0.45), and no difference in parietal rTMS effect in the novel and practiced condition (F(1.18) = 0.23; p = 0.64)

#### Summary

Reaction time was reduced and accuracy increased in the practiced condition, compared to the novel condition, for both targets and non-targets. Prefrontal rTMS effects on target accuracy were significantly lower in the practiced condition than in the novel condition. Parietal rTMS effects on target accuracy were not significantly different in the practiced and novel condition. Both prefrontal and parietal rTMS did not affect reaction times, nor did they affect accuracy results for non-targets.

## Discussion

We used rTMS, guided by fMRI, to test the hypothesis that practice in a consistent task environment reduces involvement of the left prefrontal and left parietal regions of the brain. To do this, we compared the degree to which rTMS applied at these regions affected performance of a Sternberg task with novel and practiced target-sets. 

The main finding of our study is that the degree to which prefrontal rTMS reduced subjects’ accuracy in detecting targets was significantly lower for practiced target-sets, than for novel sets. The interference resulting from parietal rTMS was not significantly different between novel and practiced target-sets. Additional findings included that neither prefrontal nor parietal rTMS interfered with non-target accuracy. Also, neither prefrontal nor parietal rTMS interfered with reaction times. Our results support the hypothesis that practice reduces the involvement of left prefrontal cortex in task execution [1,24,25]. 

Our rTMS results for the novel condition are in line with previous studies, which have consistently demonstrated interference effects of prefrontal rTMS for verbal working memory [14,15], as well as spatial working memory [26,27]. Our fMRI results indicated that practiced target-sets evoked less activity than novel target-sets in an extensive network of brain regions, including the left prefrontal cortex and left inferior parietal cortex—the rTMS focus sites. This result reproduces previous imaging results with this paradigm [8,28],[9] as well as with other paradigms [5,6,10,11]. FMRI results also confirmed that the individually selected rTMS focus sites were less activated after practice. 

With regard to parietal involvement, we found no evidence that it diminished after practice: rTMS interference effects were not different in the novel and practiced condition. This could be viewed as surprising, in light of the fact that fMRI studies have consistently shown reduced activity in parietal regions after practice, just as in prefrontal regions [29]. Possibly, the reduced activity in parietal cortex after practice is related to increased processing efficiency, without reduction in involvement. It has to be noted however, that the effect of parietal rTMS was relatively small in both the novel and practiced conditions. This is in line with previous studies that have demonstrated strong effects in prefrontal cortex, but negligible effects in parietal cortex for non-spatial cognitive tasks [27,30]. If parietal interference effects could be demonstrated, they were specific for spatial tasks [31,32]. There could be several explanations for the lower rTMS effect in parietal cortex. First, it could be that the left parietal cortex is indeed less crucial for verbal task performance then the left prefrontal cortex. A second reason could be that the left parietal cortex is part of a more extensive brain network, in which interference with a single “node”—as happens in rTMS—is insufficient to disrupt performance [30]. This is in line with the fact that our fMRI results showed bilateral parietal activity, while prefrontal activity was predominantly left lateralized. 

Our study yielded several additional findings. First, interference from rTMS was only detectable for targets, and not for non-targets. Second, rTMS interference was measurable for accuracy, but not for reaction times. This second finding replicates previously published effects of rTMS in delayed cognitive tasks [12,14]. The specific effect of prefrontal rTMS on target accuracy indicates that rTMS did not interfere with probe processing, as that would have affected non-target probes as well as targets. Similarly, it indicates that rTMS did not interfere with processes related to attention, motor execution, or inhibition, as these would also have affected non-targets and not just accuracy but also reaction time. 

One mechanism that is in line with all our findings is that left prefrontal rTMS temporarily disturbs access to the maintained target-set. Such an effect would cause target-probes to be mistaken for non-targets, while non-targets would not be affected. This is what we observed in our experiment. Reaction times would also not be affected, and this we also observed. This mechanism would also be in line with a previously published finding that the regions showing reduced activity in the practiced condition of a Sternberg paradigm are predominantly related to encoding and maintenance of the target-set [9]. 

Previously proposed mechanisms of practice either suggest that practice improves the involved processes themselves (‘process improvement mechanism’, [33-35]), or that they are replaced by other, more efficient processes (‘process switching mechanism’), either deliberately, or involuntary as the new process strengthens [36]. Two important process switching mechanisms that are capable of explaining many behavioral practice effects are ‘Item based learning’ and ‘Category Comparison Strategy’. The Item-based learning mechanism, also referred to as automatic processing, proposes that with practice we learn to associate a particular stimulus with a particular response [3,37,38]. Thus, after practice, a response can be selected without using the target-set. The Category Comparison Strategy argues that with practice we learn new categories. After practice, responses can be selected by assigning a category to a probe, also without use of target information. Importantly, for the task applied in this study it has been noted previously that there is no discernible difference between the two process switching mechanisms (Logan et al, 1988).

Our results appear to support process switching mechanisms, as the finding that prefrontal cortex appears to be less involved after practice, suggests a process switch. Additionally, the finding that TMS only interfered with target detection in the novel condition, suggests that the regions that the targeted prefrontal region was involved in either maintenance or comparison of targets, and that it is specifically this process that becomes obsolete after practice. 

Our results do not appear to support mechanisms that are based on process improvement. According to these mechanisms, one should not expect a large difference in interference effects of TMS for novel and practiced performance, as the same brain regions should be involved in novel as well as in practices performance. Yet this is not what we found for the left prefrontal cortex.’

Our findings are in line with the notion that practice can play a pivotal role in performance of complex cognitive tasks that at first sight may not appear to have a consistent environment. Complex cognitive tasks can however in many occasions be organized as a combination of many simple consistent as well as well as varied mapping sub-tasks, At first, practice may only have a direct effect on the consistent mapping sub-tasks, as performance will improve and involvement of the prefrontal cortex will reduce after practice. In turn, as there are more resources available after practice, individuals can however also show improved performance on the varied mapping sub-tasks, Thus performance on every level of the complex task may improve due to practice. This interpretation is also in line with a previous study that demonstrated that the level to which brain activity is reduced after practice is a predictor of how a subject will perform on a second unpracticed task done simultaneously [8]. 

Several methodological considerations should be taken into account. Subjects reported that they were aware of the different effects of sham and rTMS coils. This could have affected our results. However, the fact that both non-target accuracy and reaction times were similar for sham and rTMS sessions argues against the occurrence of expectation effects. The differential effects of prefrontal and parietal rTMS further support the notion that the effects of prefrontal rTMS were indeed due to electromagnetic disruption. In the present study we selected rTMS target regions based on individual activity maps rather than group based activity maps, to ensure that we targeted individual "hotspots" as closely as possible. An unanticipated result in the present study is the large variability in individual parietal hotspots ranging from inferior to superior cortices. It is possible that the hotspot may not have reflected the same underlying function, and this could have affected our results for the parietal rTMS sessions. 

In conclusion, our study showed that rTMS on left prefrontal and left parietal cortex reduced accuracy specifically for novel target-sets. Only for prefrontal rTMS, the rTMS interference effect was significantly smaller for practiced target-sets. These findings indicate that practiced performance relies less on involvement of the left prefrontal cortex. The findings support the notion that in novel performance, left prefrontal cortex is predominantly involved in maintenance comparison with the target-set, and suggest that consistent practice allows individuals to select a response without referring to the target-set. Our results also support the notion that consistent practice allows for redistribution of limited maintenance resources, and can thus be crucial in performance of complex cognitive tasks.
